# Improved detection of cerebral microbleeds in Alzheimer’s Disease using 7T susceptibility weighted imaging

**DOI:** 10.1002/alz.089906

**Published:** 2025-01-09

**Authors:** Laya Ashouri

**Affiliations:** ^1^ Urmia University of Medical Science, Urmia, West Azarbayjan Iran (Islamic Republic of)

## Abstract

**Background:**

Cerebral microbleeds (CMBs) are small, round aggregations of hemosiderin‐laden macrophages that indicate leakage of blood products from cerebral vessels damaged by β‐amyloid‐40 (Aβ), which is the basis of cerebral amyloid angiopathy (CAA). Pathology studies have demonstrated a high correlation between CAA and AD, both amyloid‐related pathologies. CMBs can be visualized commonly using T2*‐weighted MRI, as small foci of decreased signal intensity. The increased magnetic field strength at 7 Tesla (7T) MRI results in substantially enhanced sensitivity to variations in magnetic susceptibility. The more robust SWI at 7T MRI proves especially advantageous in the detection of CMBs, which are small hemorrhagic lesions distinguished by subtle alterations in magnetic susceptibility. In this study, we used the CLEAR SWI technique at 7T MRI to improve the visualization of CMBs that might be missed or undetectable with lower field strengths to better understand the role of CAA in mild cognitive impairment (MCI) and AD patients.

**Method:**

Imaging with four MCI, two AD, and six healthy subjects (73 ± 6 years old) was performed using a 7T MRI scanner. CMBs were defined as small hypointense lesions within the brain parenchyma on the SWI images. The microbleeds were categorized into two groups according to their locations: non‐lobar region and lobar. Calcifications of the basal ganglia and deep cerebellar nuclei were considered as normal findings. The analysis was performed to evaluate the associations between the number of CMBs with the patient’s Aβ PET SUVR levels.

**Result:**

Our results showed that out of 51 observed CMBs in six MCI/AD patients 48 (94%) CMBs were lobar and 3 (6%) non‐lobar. A total of four CMBs were found in three AD/MCI patients. A total of three CMBs (one lobar and 2 non‐lobar) were observed in six healthy participants. A significant correlation was found between the number of lobar CMBs and the Aβ PET SUVR levels in AD/MCI patients.

**Conclusion:**

CMBs have attracted attention in Amyloid immunization therapy trials in AD, therefore improved visualization of CMBs with CLEAR SWI sequences at 7T MRI may contribute to more accurate identification of CMBs, and better understanding of their role in MCI and AD.

